# New Framework for Dynamic Water Environmental Capacity Estimation Integrating the Hydro-Environmental Model and Load–Duration Curve Method—A Case Study in Data-Scarce Luanhe River Basin

**DOI:** 10.3390/ijerph19148389

**Published:** 2022-07-09

**Authors:** Huiyu Jin, Wanqi Chen, Zhenghong Zhao, Jiajia Wang, Weichun Ma

**Affiliations:** 1 Department of Environmental Science and Engineering, Fudan University, Shanghai 200433, China; 20110740007@fudan.edu.cn (H.J.); wqchen9887@163.com (W.C.); 20210740091@fudan.edu.cn (Z.Z.); 19210740014@fudan.edu.cn (J.W.); 2 Institute of Eco-Chongming (IEC), No. 3663 Northern Zhongshan Road, Shanghai 200062, China; 3 Shanghai Key Laboratory of Policy Simulation and Assessment for Ecology and Environment Governance, Shanghai 201804, China; 4 Institute of Digitalized Sustainable Transformation, Fudan University, Shanghai 200433, China; 5 Institute for Big Data (IBD), Fudan University, Shanghai 200433, China

**Keywords:** water environmental capacity (WEC), SWAT, load-duration curve (LDC), pollution load distribution, water quality

## Abstract

A better understanding of river capacity for contaminants (i.e., water environmental capacity, WEC) is essential for the reasonable utilization of water resources, providing government’s with guidance about sewage discharge management, and allocating investments for pollutant reduction. This paper applied a new framework integrating a modified hydro-environmental model, Soil and Water Assessment Tool (SWAT) model, and load–duration curve (LDC) method for the dynamic estimation of the NH_3_-N WEC of the data-scarce Luanhe River basin in China. The impact mechanisms of hydrological and temperature conditions on WEC are discussed. We found that 77% of the WEC was concentrated in 40% hydrological guarantee flow rates. While the increasing flow velocity promoted the pollutant decay rate, it shortened its traveling time in streams, eventually reducing the river WEC. The results suggest that the integrated framework combined the merits of the traditional LDC method and the mechanism model. Thus, the integrated framework dynamically presents the WEC’s spatiotemporal distribution under different hydrological regimes with fewer data. It can also be applied in multi-segment rivers to help managers identify hot spots for fragile water environmental regions and periods at the basin scale.

## 1. Introduction

The rapid development of urbanization and the social economy has caused river quality degradation in developing countries [1,2]. With the effective control of oxygen-consuming organic pollutants, ammonia nitrogen (NH_3_-N), one of the most common aquatic contaminants responsible for eutrophication, has been included as a constraint indicator of total pollutant discharge control since 2011 (Twelfth Five-year Plan in China). Furthermore, in the Fourteenth Five-year Plan in China, the government put forward a target to reduce the total NH_3_-N discharge by more than 8% between 2020 and 2025. Under the overall reduction objective, there is an urgent requirement for scientific planning of allowable load allocation to combine the reduction target and local water quality standards throughout the country [3]. Water environmental capacity (WEC) is one of the most effective strategies currently adopted in domestic environmental management [4,5,6]. It plays a similar role to total maximum daily loads (TMDLs) [7,8,9], carrying capacity [10], assimilative capacity [11,12], and so on, which reflects the maximum abilities of a water body to accommodate pollutants under the limitation of target water quality [13]. According to the Chinese Academy for Environmental Planning (CAEP) [4], for better management of pollutant discharge, WECs have been further categorized: ideal WEC, actual WEC, and remnant WEC. Ideal WEC is a natural property of water bodies, representing the self-cleaning capacity of a water body through dilution and assimilation processes. It is quantified by the maximum allowable loads, consisting of point and non-point source pollutants. Because non-point source pollution (e.g., agricultural or urban surface runoff) is challenging to control, CAEP proposed the concept of actual WEC, representing the maximum allowable loads for point source pollutants. Actual WEC is calculated by the difference between the ideal WEC and non-point source pollution loads. It is mainly used by the government to regulate and control wastewater discharge from local industries and large-scaled livestock breeding farms. Meanwhile, remnant WEC is the carrying capacity left after deducting all existing point source pollutions from the actual WEC. It reflects the local pollution state, and whether the water body can accommodate more pollutants. Therefore, both actual and remnant WECs are calculated based on the ideal WEC. With the estimation of ideal WEC, the total maximum allowable loads can be quantified and appropriately allocated between the different regions to coordinate socioeconomic development and aquatic environmental protection. Combining the investigation of synchronous discharges of two pollution types, the other WECs can be further determined.

To determine WEC, many methods have emerged based on mechanistic water quality models [14,15] and the system-optimized model [16,17]. However, these methods are associated with abundant iterative trials and tedious calibrations, which can therefore hardly be popularized nationwide. In addition to the complicated approaches, the Environmental Protection Agency (EPA) provided a load–duration curve (LDC) method, which is straightforward for estimating the loading capacity [18]. The core of the LDC method is to establish the flow cumulative frequency curve (i.e., flow duration curve (FDC)) based on the historical runoff data and multiply it by the water quality standards to generate the maximum allowable loads in each flow regime (herein similar to ideal WEC). The LDC method can easily be used in any river section and easily connects the flow variability with the number of allowable loads [19,20]. However, it does not consider the transport and transformation mechanisms after the pollutants load into the river [21]. Thus, it would be deficient if other factors, e.g., temperature and flow velocity, also greatly impact the pollutant’s fate. The method based on the steady-state water quality model is consequently preferred for estimating the river WEC because it considers the pollutants’ decay processes with lower computing resources and data needs [4,21]. Currently, the domestic studies of WECs management were mainly based on the guidance from CAEP [4], taking a 90% hydrological guarantee flow rate (i.e., low flow condition) or the driest monthly average discharge in the last 10 years of statistics as the design conditions for WEC estimation [3,22]. Li et al. [5] fond that the annual NH_3_-N WEC calculated under 90% guarantee flow rate was half of the result under a 70% guarantee flow rate in the Zhangweinan subbasin. Lacking the consideration of the spatial-temporal heterogeneity of flow conditions is the main reason for the uncertainty of WEC estimation. Furthermore, a long-term hydrological dataset is hard to collect, especially for data-scarce regions, as even the determination of a guarantee flow rate has to reference the adjacent river net. The lack of hydrological monitoring causes difficulties in water quality protection [23,24]. In addition to flow rate, another key parameter for WEC estimation is the integrated degradation coefficient (k), which determines the decay rate of the contaminant in the river channel. In previous studies based on steady-state model, k was usually considered to be a constant [3,12,25]. Actually, it has been confirmed that k is simultaneously affected by flow velocity and water temperature [24,25,26,27]. Even for some mechanism water quality models, Qual2K, for instance, only takes into account temperature correction for k whilst ignoring the effect of velocity [28]. Zhang et al. [27] calculated the WEC in Xiaohe river basin based on MIKE11 model, while neglecting the effects of temperature variation on k. Therefore, there is an urgent need for the localized parametrization and quantification of effect magnitudes from key factors on k. After addressing the essential roles played by these factors, a further estimation of the ideal WEC at different spatiotemporal scales can be taken to fill the gap that makes the steady-state model inapplicable under dynamic river conditions.

The methods of ideal WEC can be classified as head-control and end-control according to the pollutant control strategies in the steady-state model. Both strategies assume the pollutants’ input at the uppermost river segment. The difference is whether the water quality throughout the traveling period obeys the criterial level setting from the uppermost section (head-control) or is allowed to overshoot briefly—as long as it achieves the standard by the downstream section (end-control) [25]. They, respectively, represent the lowest and highest limits of pollutant discharging in WEC management. These two strategies have applicability, for example, as head-control is more beneficial for aquatic community remediation (e.g., ecological preservation area) [24] and end-control is more suitable for rivers with top priority for deterioration control [29]. The safety margins for most general water bodies lay between these two levels. Developing a compromising method that is more suitable for the general river basin in developing countries and regions is necessary.

This research took the mainstream Luanhe river basin as a case study, providing an integrated ideal WEC estimation framework, combining Soil and Water Assessment Tool (SWAT) [30,31] to apply in this data-scarce basin. The main research aims are:

(1) Provide a more applicable method for in-stream WEC calculation in developing regions based on a modified pollutant control strategy;
(2) Discuss the impact mechanisms of hydrological and temperature conditions on WEC in detail and establish the real-time responses of WEC to these factors;
(3) Integrate the modified WEC estimation method and the real-time response process to develop a hydro-environmental model for dynamic WEC estimation;
(4) Combine the SWAT model and LDC method to present a spatiotemporal distribution of WEC under different hydrological regimes; identify the hot spots of fragile regions and periods; and provide suggestions for managers about the collaborative pollutant control at a basin scale.

## 2. Materials and Methods

### 2.1. Study Site

**Figure 1:**
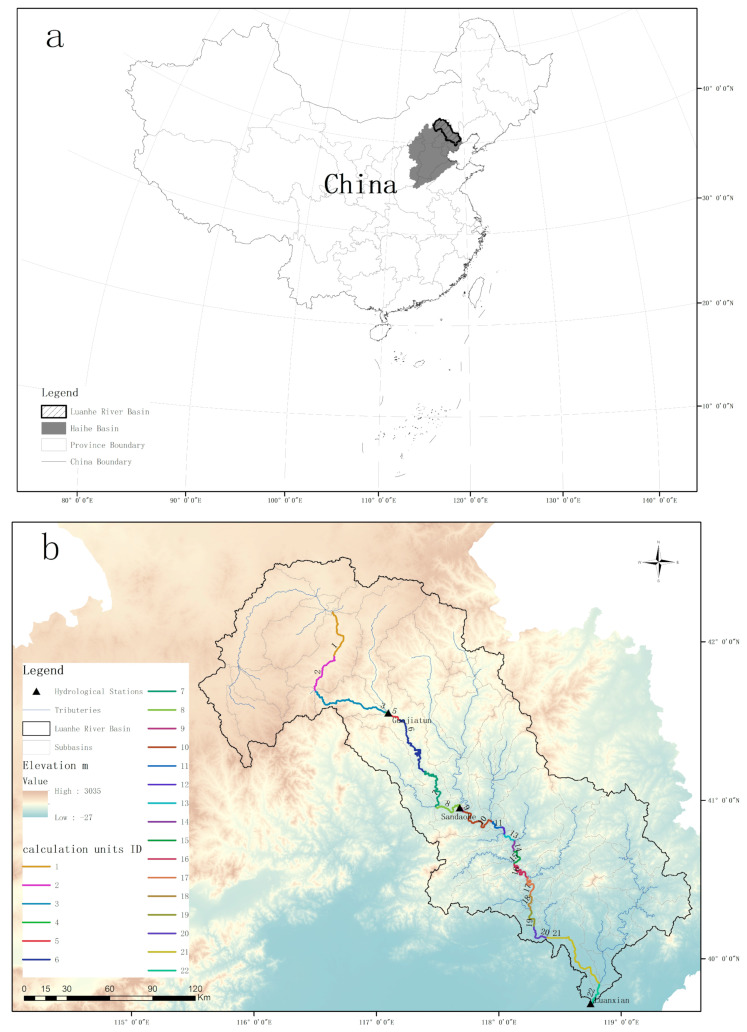

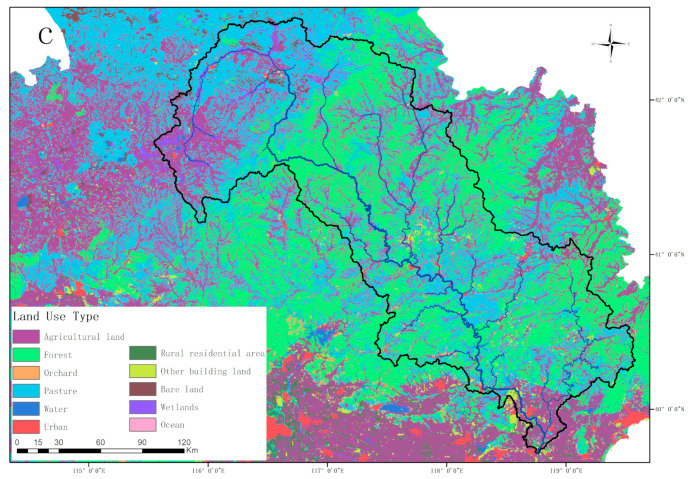
Study area. (**a**) Location of Luanhe and Haihe basins in China; (**b**) topography, river streams, and hydrological stations in the study basin; the mainstream of Luanhe is divided into 22 calculation units from upstream to downstream, the classification number in Figure (**b**) was the sequence number of calculation unit ID, and WECs were estimated based on each unit separately (these calculation units were labeled as Reach 1–Reach 22 in the following context); (**c**) land-use types in 2015 from China resource and environmental data platform.

Luanhe River basin (115°27′ E–119°56′ E, 39.43° N–42°41′ N) is located in the northeastern part of the North China Plain, which is an arid to semi-arid region with a total area of 42,641 km^2^. It is one of the sub-basins of the Haihe—the largest basin of northern China, which covers the Beijing–Tianjin–Hebei urban agglomeration. The upper reaches of the Luanhe river basin are plateau areas with a large variety of elevations, and the midstream is in an incised valley in a hilly region. The downstream is a piedmont plain, which is greatly affected by human activities [32]. The mainstream Luanhe River flows through 17 subbasins from the northwestern to northeastern area. The average total annual precipitation and temperature from 2006 to 2019 were 445.87 ± 107.6 mm and 7.5 ± 4.54 °C, respectively, with nonnegligible spatiotemporal heterogeneity. From the 2015 land-use distribution, forests and grassland covered 39.51% and 30.26%, respectively, of the whole river basin, and agricultural land occupying 22.44%. The remaining land types were building land, water, and bare land. More regional details are shown in Figure 1.

### 2.2. Integrated Framework for Dynamic Water Environmental Capacity Estimation

**Figure 2:**
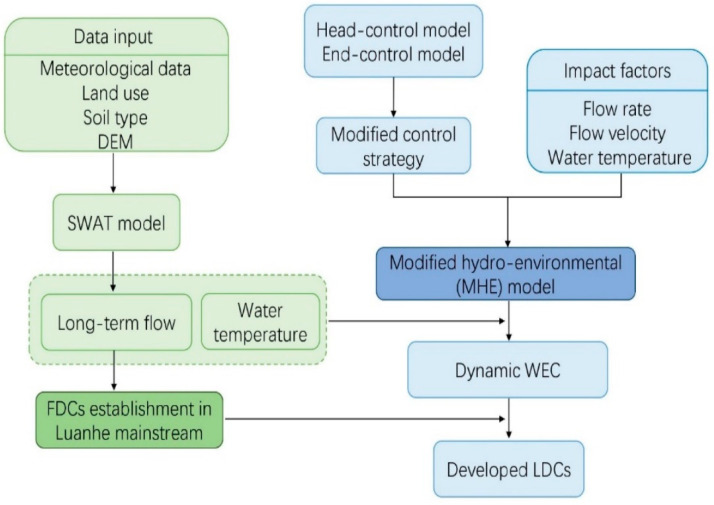
Flowchart of integrated estimation method based on a modified hydro-environmental (MHE) model and load–duration curve method.

The framework consists of two key steps—the modified hydro-environmental (MHE) model establishment [25] and the LDC development. The core of the MHE model is the one-dimensional water quality model and a modified pollutant control strategy. To consider a dynamic perspective, the real-time responses of WEC to various hydrological and temperature conditions were established. Due to the lack of observed hydrological data in mainstream Luanhe, the SWAT model was used for the development of FDCs in each subbasin, which provided the flow variabilities of the river basins. Combining the MHE model and FDCs, developed LDCs were established on the basin scale to discuss the spatial-temporal distribution of WECs under different hydrological regimes. The flowchart of the integrated framework is shown in Figure 2.

#### 2.2.1. SWAT Model Setup and Evaluation

The low availability of flow data in the Luanhe River basin is an obstacle in establishing FDCs and continuous long-term WEC research that can be overcome with hydrological modeling. SWAT is a semi-distributed, process-based model for time-continuous hydrological processes and water quality simulation [20]. It is widely used for runoff and non-point source pollution predictions under different management practices, weather, land use, and soil conditions. Because of its powerful physically based mechanism, the SWAT model is desirably applied in data-scarce or ungauged areas. The hydrological SWAT process is formed with land and channel modules and driven by water balance factors, including precipitation, infiltration, runoff, groundwater returns, channel movement loss, evapotranspiration, etc. [33]. Spatially explicit DEMs, land use, and soil and climate data are required for the SWAT model. A 2015 land-use map and topography data of the study basin were obtained from the China resource and environmental data platform. Soil data were obtained from the Harmonized World Soil Database (HWSD) [34,35], and all spatial data were resampled to 200 m with the nearest-neighbor method in ArcGIS. Input climate data were from the China Meteorological Assimilation Datasets (CMAD) [34,36,37,38,39,40,41], including daily precipitation, temperature, wind speed, relative humidity, and solar radiation. The missing climate data were replaced through the weather generator (WGN) within the SWAT model.

The monthly average flow data of two hydrological monitoring gauges (Wulongji and Luanxian) from 2012 to 2016 were used for model validation (validation data were provided by the China Ministry of Ecology and Environment). The modeling period of SWAT depends on the input climate data (CMAD), which were from 2008 to 2016. As a result, we set 2008–2011 as the warm-up period and 2012–2014 and 2015–2016 as the calibration and validation periods, respectively. The performance of flow estimation was analyzed through two indexes, Nash and Sutcliffe efficiency (NSE) [42] and R^2^ (correlation coefficient) [43]. Considering the complicated mechanisms and the large number of parameters involved during simulation processes, SWAT Calibration and Uncertainty Programs (SWAT-CUP) was utilized for sensitivity analysis and the best combination searching of parameters. Furthermore, we used the Sequential Uncertainty Fitting version 2 (SUFI-2) algorithm in SWAT-CUP as a calibration algorithm [44]. Based on 5 years of monthly average flow data simulated through SWAT, the FDCs in each sub-watershed were created in the Luanhe mainstream.

#### 2.2.2. Modified Hydro-Environmental Model Establishment

##### Water Environmental Capacity Calculation

Because the lateral length of the sewage mixing process is far less than the river length, a one-dimensional water quality model is applicable here [21]. It contains a flow movement module, which is described by the Saint-Venant equations, as shown in Equations (1) and (2), and an advection–dispersion module in Equation (3) [21,45]. Equation (4) represents the decay process of river pollutants in the one-dimension steady system.

(1) $$\frac{\partial Q}{\partial x}+\frac{\partial A}{\partial t}=0$$

(2) $$\frac{\partial Q}{\partial t}+\frac{\partial }{\partial x}\left(\frac{Q^{2}}{A}\right)+gA\frac{\partial Z}{\partial x}+\frac{gQ}{h^{2}}\frac{\left|Q\right|}{RA}=0$$

(3) $$\frac{\partial AC}{\partial t}+\frac{\partial QC}{\partial x}=\frac{\partial }{\partial x}\left(E_{x}\frac{\partial AC}{\partial x}\right)+AS+Af\left(C,t\right)$$

(4) $$u\frac{\partial C}{\partial x}=-kC\;$$

where *Q* is the flow rate (m^3^/s); *A* is the cross-sectional area (m^2^); *x* (m) is the distance of water flow from the headstream through travel time *t* (s); g is the gravitational acceleration in m/s^2^; *Z* is the water level in m; h is the Chezy coefficient (m^1/2^/s); and *R* is the hydraulic radius in m. In the advection–dispersion module, *C* is the concentration of reaction pollutants in mg/L; *Ex* is the diffusion coefficient in m^2^/s; *S* refers to the source/sink terms in (mg/L/s); *f* is the reaction term for NH_3_-N; and *u* is the average flow velocity (m/s).

**Figure 3:**
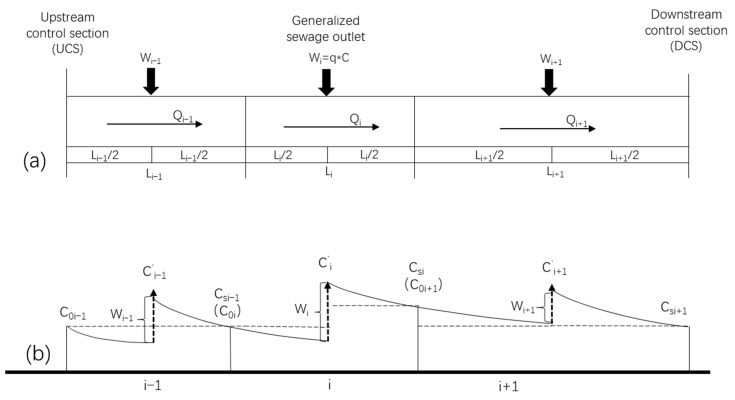
The diagram of the stream sections’ generalized sewage outlets (**a**) and pollutant degradation processes (**b**).

Based on this decay regulation, we developed a hydro-environmental model, as illustrated in Figure 3. We generalized a pollutant outlet involving point and non-point sources discharged in the middle of each calculated unit rather than the head section such as in the other two strategies mentioned above. The contaminants, therefore, experienced different decay processes before and after the load inputs. The first half of the stream follows the quality standard set in the head section; the end-control is carried out in the second half of the stream. The processes are described in Equations (5) and (6):

(5) $$C_{i}^{\prime }=\frac{C_{0i}e^{(-\frac{k_{i}\frac{1}{2}L_{i}}{86.4u_{i}})}Q_{i}+W_{i}}{Q_{i}+q_{i}}$$

(6) $$C_{si}=C_{i}^{\prime }e^{\left(-\frac{k_{i}\frac{1}{2}L_{i}}{86.4u_{i}}\right)}$$

In Equation (5), *i* stands for the number of calculated units in the Luanhe mainstream, *i* = 1, 2, …, 22; $L_{i}$ is the length of the calculated unit in km; $u_{i}$ is the average flow velocity (m/s); $q_{i}$ is the sewage discharge in m^3^/s; $Q_{i}$ is the river discharge of each unit in m^3^/s; assuming that the pollutants from the sewage outlets are fully mixed with the river water at the moment of discharge into the channel, the concentration of NH_3_-N at the discharge outlet is $C_{i}^{\prime }$; $C_{0}$ and $C_{si}$ refer to NH_3_-N concentrations at the upstream and down control cross-section in each calculated unit, all in mg/L; when $C_{si}$ is equal to water quality criteria, $W_{i}$ is the water environmental capacity at each calculated unit (kg/d), representing the maximum allowable loads discharged by the sewage outlet. By combining two equations, $W_{i}$ can be derived in Equation (7). Because the calculated result of $W_{i}$ is in g/s, a conversation factor (86.4) is necessary to change it to kg/d. Additionally, the maximum allowable loads in Luanhe mainstream is the sum of WEC in 22 calculated units.

(7) $$W_{i}=\left[Q_{i}C_{si}e^{k_{i}\times \frac{L_{i}}{86.4\times 2u_{i}}}-Q_{i}C_{0i}e^{-k_{i}\times \frac{L_{i}}{86.4\times 2u_{i}}}\right]\times 86.4$$

##### Model Parameters Determinations

Integrated degradation coefficient (*k*, d^−1^) and flow velocity (*u*) are crucial parameters in WEC calculation. From Equation (4), *k* can be determined through equation: $k=86.4\text{ln}\left(\frac{C_{0}}{C_{1}}\right)\left(\frac{u}{L}\right).$ In the one-dimensional model, decay rate is quite sensitive to water temperature. According to the Arrhenius equation [46], the temperature adjustment *k* can be presented as:

(8) $$k_{2}=k_{1}\times \theta^{\left(T_{2}-T_{1}\right)}$$

where *k*_1_ is the degradation coefficient under a referenced temperature, which is usually set at 20 °C; *T*_2_ is the actual water temperature; *θ* is the empirical parameter and should be calibrated. Taking the log transform of both sides of the equation, the degradation rate and temperature can be solved with the equation: $T_{2}=\frac{ln\frac{k_{2}}{k_{1}}}{ln\theta }+T_{1}$. If we take $\frac{1}{ln\theta }$ as a gradient under the linear regression between *T*_2_ and $\ln\frac{k_{2}}{k_{1}}$, *θ* can be calibrated with a few measured *k*’s in different water temperatures. Therefore, combined with the above equations, the dynamic *k* can be finally decided with Equation (9)

(9) $$k=86.4a{\left(\frac{u}{L}\right)}^{b+1}\theta^{\left(T_{i}-20\right)}$$

where *a* and *b* are parameters that can be directly calibrated in the equation by several observations or based on the relationship $\ln\frac{C_{0}}{C_{1}}=a{\left(\frac{u}{L}\right)}^{b}$ [24] under 20 °C. During the calibration of *k*, the selected river section must be relatively straight with a stable flow rate, avoiding sewage outlets, tributaries into the river, dams, and other blocking buildings. Meanwhile, the synchronous monitoring of the water quality and flow and velocity of up- and downstream cross-sections should be fully provided. In this research, the daily water temperature, hydrological, and water quality data used for decay rate calibration were provided by the China Ministry of Ecology and Environment.

The other crucial factor relating to the WEC calculation is velocity. Leopold and Maddock [47] put forward an empirical power exponent formula to describe the relationship between flow rate and velocity:

(10) $$u=aQ^{b}$$

where *u* represents flow velocity (m/s); *Q* is the flow rate (m^3^/s); *a* and *b* are empirical coefficients calibrated by observed flow rate and velocity data, respectively. The local hydrological yearbook in 2015 [48] provided synchronous monitoring flow rate and velocity data in Luanhe mainstream, which was used to calibrate these empirical coefficients.

## 3. Results

### 3.1. Historical Flow Estimation Based on SWAT Model and Traditional LDC Creating

**Figure 4:**
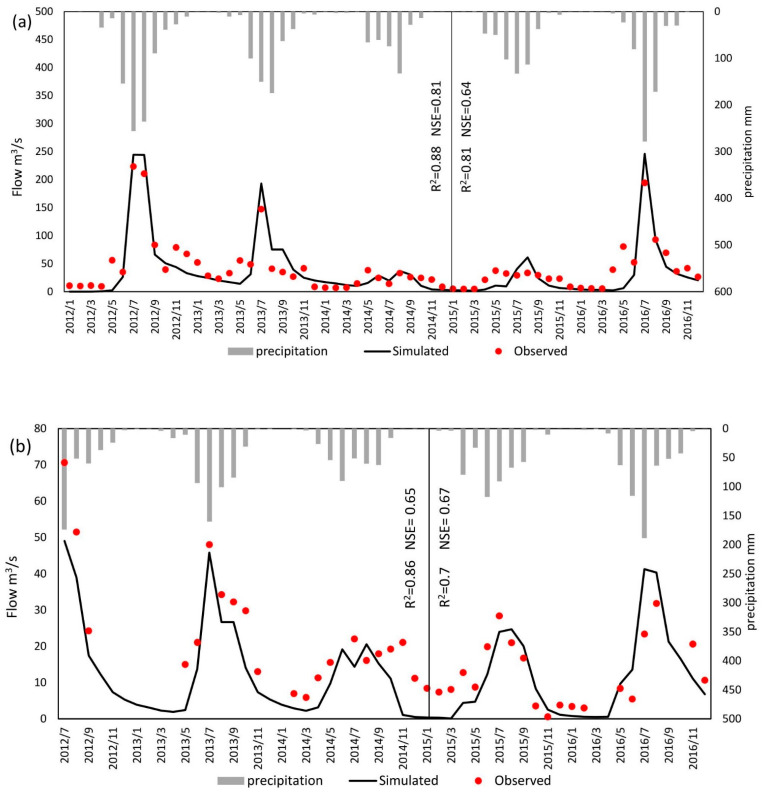
Calibration and validation results of streamflow from 2012 to 2016 in (**a**) Luanxian and (**b**) Wulongji stations.

**Figure 5:**
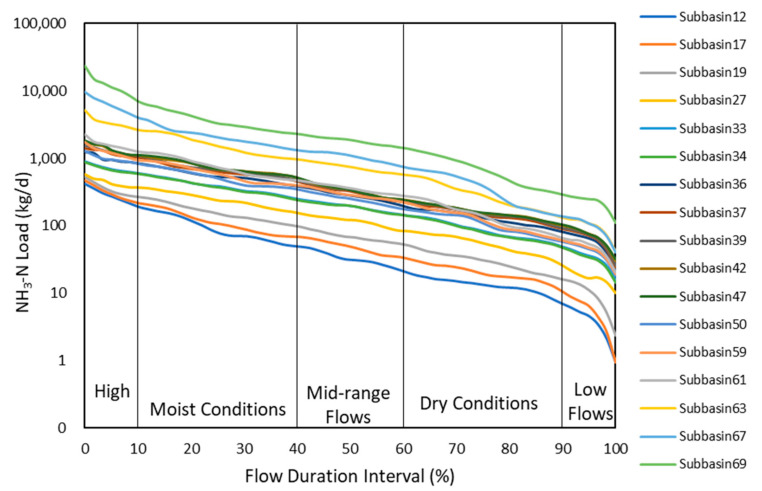
Traditional load–duration curves in 22 subbasins of mainstream Luanhe.

Depending on the time range of input climate dataset, the modeling period of SWAT was from 2008 to 2016. Additionally, because the validation hydrological datasets were from 2012 to 2016, the calibration and validation periods of the model were set from 2012 to 2014 and 2015 to 2016, respectively, and 2008–2011 was set as warm-up period. The whole Luanhe river basin was divided into 69 subbasins. According to Moriasi et al. [42], NSE and R^2^ indexes above 0.5 were acceptable for distributed process-based hydrological models. The performance ratings of streamflow predictions are presented in Table 1. The performance was “Good” or “Adequate” for both stations during both periods. Figure 4 demonstrated that July–September was the rainy season in the studied watershed. Meanwhile, 2012, 2013, and 2016 were wetter than the other years, with a streamflow of more than 100 m^3^/s during wet periods. From Geng et al. [49], precipitation is the primary regulation of streamflow in the Luanhe river basin. This is reflected in Figure 4, as variations of river runoff were highly related to monthly accumulated precipitation. Continuously heavy rainfall was the main trigger for the runoff peak value. According to USEPA [18], FDCs can generally be divided into five flow duration intervals (FDIs): 0–10% is defined as the high flow interval (HFI); 10–40% is the moist conditions interval (MCI); 40–60% (MFI) is the mid-range flow interval; 60–90% and 90–100% are the dry conditions interval (DCI) and low flow interval (LFI), respectively (Figure 5). Combined with the long-term river runoff simulated through SWAT and each subbasin’s allowable ammonia nitrogen concentration, we established LDCs for Luanhe mainstream. From Figure 5, there was an overall gradual increase in permitted load discharges from upstream to the outlet, and these variations were dominated by streamflow conditions and water quality standards.

### 3.2. The Calibration of Integrated Degradation Coefficient and Relevant Influencing Factors

Water temperature (*T*) and flow velocity (*u*) are two main uncertainties in the decay rate estimation [24,50]. However, few researchers quantified the magnitudes of these factors. Common WEC estimations directly reference the value from laboratory research or obtain the recommended values from national guidelines [4] lacking spatiotemporal anisotropy. Thus, our research calculated the decay rate, which can fluctuate with temperature and hydrological conditions based on the relationships between *k*–*T* and *k*–*u*. Furthermore, we compared the results with relevant research in the Haihe basin.

**Figure 6:**
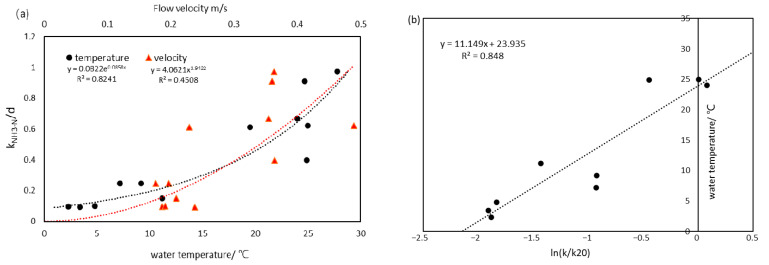
(**a**) The relationship between NH_3_-N degradation coefficient, water temperature, and flow velocity; and (**b**) the calibration of temperature coefficient θ.

**Figure 7:**
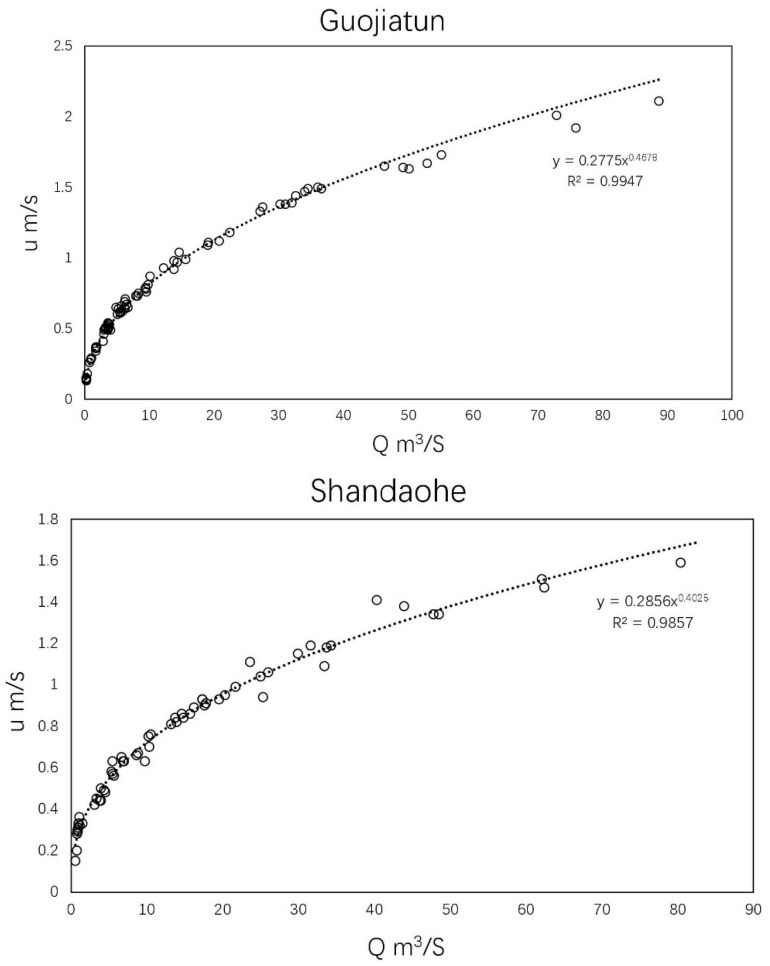

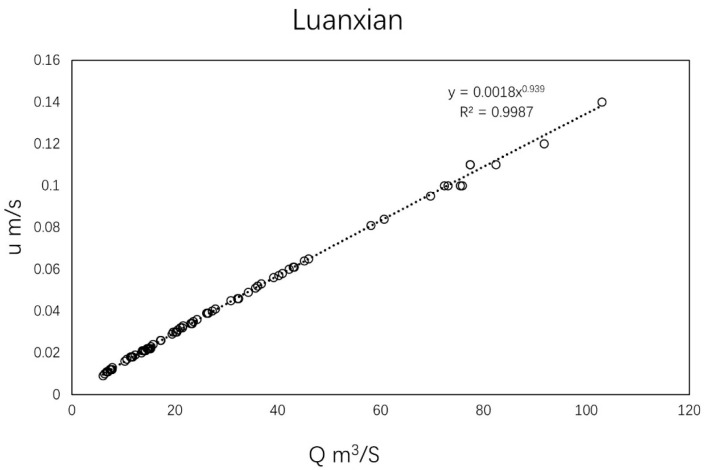
Empirical relationship between flow and velocity in three hydrological gauges of Luanhe mainstream.

Limited by the lack of synchronous water quality and daily flow observations in Luanhe, Ziya River (also belonging to the Haihe basin) was selected for the localization of *k*. Daily pollutant concentrations of the Yanjiazhuang Bridge section (YJZB), Xiahuai Town section (XHT), and Xiaojue hydrological gauge from 2014 to 2019 were used for the dynamic k measurement, according to Equation (9). The measured k ranged from 0.09 to 0.67 at 2.3~25 °C, slightly higher than the research of Shan [51] (0.025–0.521 at 5~27.5 °C). The differences may be because the latter was measured in an incubator under different light and temperature conditions without considering the impact of streamflow. The effects of flow velocity and temperature on k for NH_3_-N are further demonstrated in Figure 6a. Both factors had varying positive influences on the decay process of nutrient contaminants. Using Equations (8) and (9), *θ* was calibrated as 1.09 with an R^2^ of 0.85; *a* and *b* were calibrated as 0.074 and −0.4928, respectively. As a result, the dynamic *k* for NH_3_-N can be described in Equation (11) with an RMSE = 0.64/d and R^2^ = 0.79. Additionally, combined with the rating curves between *Q* and *u* of the monitoring gauges in Luanhe mainstream, which are shown in Figure 7, the flow velocity of Luanhe can be calculated through SWAT modeling flow rates. Localized *k* can therefore be solved.

(11) $$k=6.406{\left(\frac{u}{L}\right)}^{0.5072}1.09^{\left(T_{i}-20\right)}$$

### 3.3. The Results of the Modified Hydro-Environmental Model

**Figure 8:**
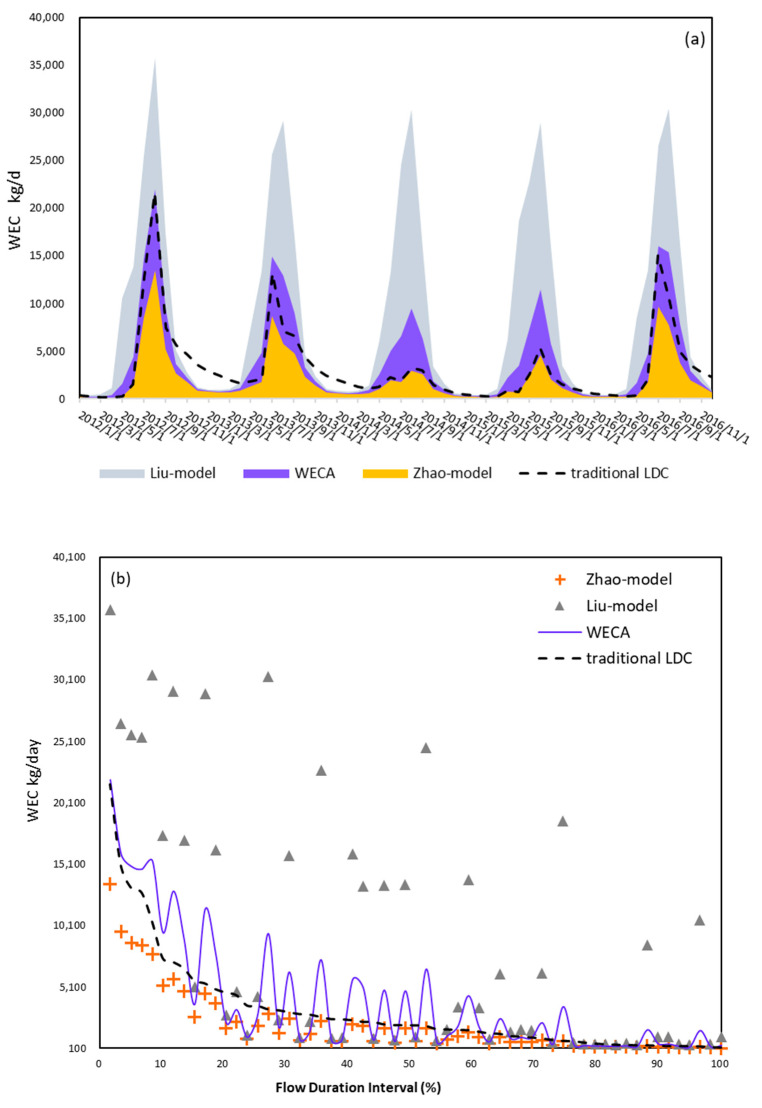

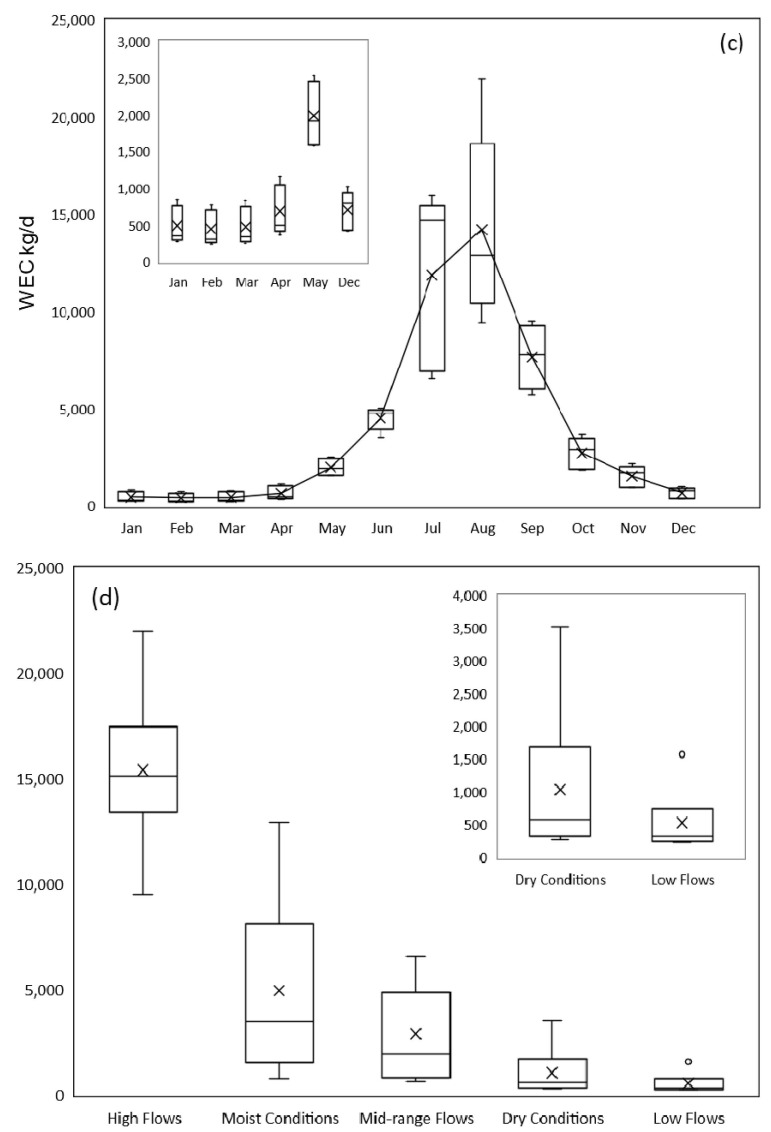
Comparison of MHE model, Liu-model (end-control model), Zhao-model (head-control model), and the traditional LDC model in the outlet subbasin under: (**a**) time series from 2012 to 2016; and (**b**) different flow regimes. (**c**,**d**) Capacity variations from the MHE model of outlet subbasin at different months and flow regimes, respectively.

There was a more significant fluctuation of load capacity by the MHE model compared with traditional LDC methods, as shown in Figure 8a,b. Under the disturbance by temperature and flow velocity, the river basin had more WECs for NH_3_-N in most high flow conditions compared with LDC. Nevertheless, runoff is still governed by the overall trend of WECs. Because of the relatively lower flow rate in 2014 and 2015, WECs in these two years were also lower than in other years. To compare the performance of MHE models, we used the head-control model by Zhao et al. [24] and the end-control model by Liu et al. [52] to study the uncertainties of WEC calculations. The three models presented similar trends during five years, higher in the summer and rainy seasons, always peaking in August. During most flow conditions, there were significant differences between the head and end control models, and the maximum difference was up to 27,500 kg/d. The MHE model results were always between the two models, but closer to the head-control. The discrepancies between the MHE and head-control models were mainly concentrated in the HFI and MCI periods and were gradually closer with decreasing flow rate. Meanwhile, the overestimation of WEC from the end-control model always existed in most flow regimes compared with the other two models.

From Figure 8c, WEC was calculated via our model in the outlet subbasin, mainly concentrated in wet seasons from July to September, which accounted for 72% of the 12-month total capacity. The average dry season WECs (between December and April of the following year) were much lower with little fluctuation, remaining stable between 400 and 700 kg/d. A dramatic expansion of the monthly average WEC appeared from June to July and declined from September after the peak of 14,250 kg/d in August. In Figure 8d, although high flow conditions only took up 10% of the cumulative frequency, it contributed 40% of the self-cleaning capacity for nutrient loads during five years, three times more than that in MFI and four times more than the sum of the capacity in DCI and LFI. Comparing the median and average values in the box plots, pronounced fluctuations were found in most hydrological condition zones except for HFI, and there was a significant WEC outlier in LFI. In addition to hydrological attributes (including runoff and flow velocity), temperature also apparently affects the capacity of river basins in each FDI.

## 4. Discussion

### 4.1. Estimation of the Water Environmental Capacity

Different pollutant control strategies may cause a significant difference in WEC determination, while few models of WEC distinguish the regional applicability when choosing implementation strategies. As shown in Figure 8, the head control strategy is much stricter because it supposes that pollutant discharge outlets are in the head of river segments and follow the water quality restriction from the head section to ensure that the whole reach consistently achieves quality criteria. Meanwhile, for end control, the quality restriction is set in the downmost section of each reach. It allows pollutant concentrations of the upper river to exceed water quality criteria as long as they reach the standard before arriving at the lower section (through dilution and assimilation processes) [29]. The stricter approach is beneficial for aquatic environmental restoration; however, it is not suitable for undeveloped regions confronting the conflict between high production requirements and aquatic environmental deterioration. A better method for WEC management at the basin scale is to comprehensively consider the current states of pollutant discharge and of the aquatic environment, including sewage treatment plant distribution, local production mode, and the urbanization level, using two control methods over the whole river basin rather than a single consideration [29]. However, this comprehensive assessment is unenforceable for data-scarce regions. Moreover, using different control strategies may cause controversy over fairness in local governments, threatening management implementation [53]. As a result, the MHE model presented a more feasible idea to fulfill in the whole river basin. Shen and Zhao [21] conjunctively used the Bayesian statistics method and a mechanism model to assess the TMDL of a river on the Eastern Shore of Virginia and compared it with the traditional LDC method. The result suggested that the LDC method underestimates because it does not fully consider the impacts of the river channel’s geomorphology and temperature conditions on contaminant transport. Furthermore, WECs calculated by the head-control model are even lower than those estimated from the traditional LDC method (Figure 8), reflecting excessively conservative features of the head-control strategy. On the contrary, the MHE model presents higher applicability and promotional value in developing regions.

### 4.2. Effects of Hydrological and Temperature Conditions on WEC

There is broad recognition of the primary regulation of runoff on pollutant loads [19,54]. For instance, the LDC put forward by the EPA establishes the maximum allowable load variation according to different flow regimes [18]. However, this method ignores the influential mechanisms and magnitudes caused by temperature and flow velocity, which also significantly affect contaminant attenuation and assimilation. Water temperature manipulates WECs for NH_3_-N, mainly by affecting the pollutant decay rate. As presented in Equation (9), under the same hydrological conditions, a 10 °C increase will double the degradation coefficients, similarly to Shen and Zhao’s finding [21]. The average monthly water temperatures at Luanxian station vary between 2.9 and 27.8 °C from 2012 to 2016; thus, ignoring temperature fluctuations results in inaccurate simulations.

The effects of streamflow velocity on WECs are mainly considered as the first-order decay rate relationship. In previous studies, rapid flow always accompanies superior hydrodynamic conditions, which benefit pollutant decay processes; this was also confirmed in our research [24,52,55]. However, for estimating WECs, the effects of the discharge rate are not only on decay rate but the spatial traveling range of pollutants from the upstream cross-section. Under the same time interval, the larger velocity, the farther pollutant travels with channel water mass. This means that under greater discharge rate, the downstream water quality will be more affected by the pollutant concentration from incoming flow, leading to a lower carrying capacity of loads discharge from downstream sewage outlets. On the other hand, the pollutant will be washed to downstream together with the rapid movement flow without adequate degradation. At the same length of river channel, slower velocity provides more time for contaminants to degrade, resulting in a higher WEC, and it can be reflected by either MHE model, head or end control model.

**Figure 9:**
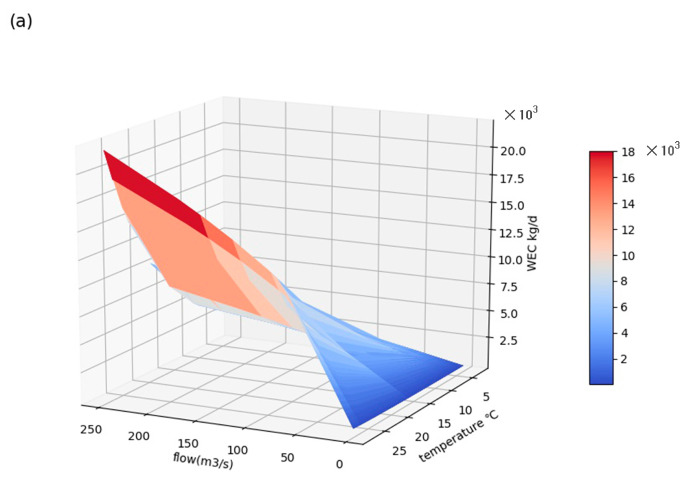

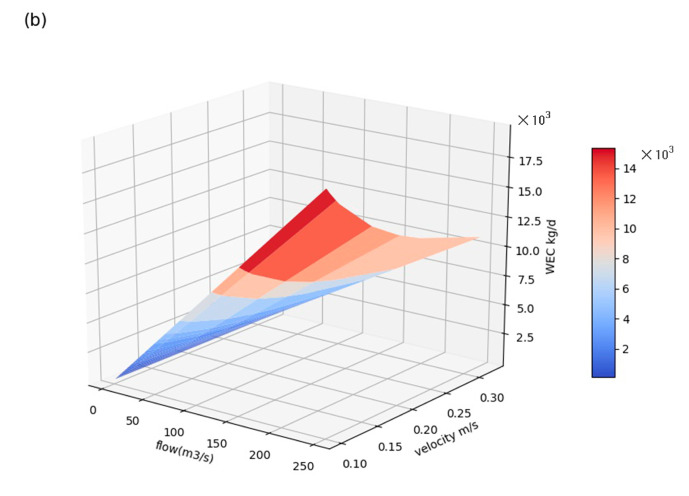
Response surfaces of WECs to (**a**) flow rate and water temperature and (**b**) flow rate and velocity under 20.

The impacts of water temperature, flow rate, and velocity on WECs in the outlet subbasin were further demonstrated through surface response diagrams in Figure 9. Under a 1 mg/L NH_3_-N concentration in the upstream background and downstream target concentration, the WECs corresponding to the temperature and flow rate of 0~30 °C and 0–250 m^3^/s are shown in Figure 9a. Thus, higher water temperature and flow rate allowed greater WECs. Specifically, a 10 °C increase was able to cause 2.4~13.85 times the increase in WECs under the unaltered hydrological conditions, and the influence of temperature would be more significant at a higher flow rate. Meanwhile, with the constant temperature of 20 °C, a 1% increase in runoff might cause a 0.05~0.53% (average increase of 0.4%) rise in the loading capacity. We also found that WECs became less sensitive to flow rate changes as the temperature decreased, especially under 5 °C. As studied by Shan [51], the integrated degradation processes for ammonia in the Haihe basin are mainly controlled by nitrifying bacteria, whose fate heavily relies on water temperature. When the temperature is <10 °C, the activity of nitrobacteria dramatically declines; at <5 °C, most nitrobacteria are in a dormant state, nearly stopping nitrification processes. During this time, WECs are mainly contributed to by dilution processes with no assimilation capacity. Figure 9b further illustrates various WECs under 20 °C, corresponding to the flow rate and velocity from 0~250 m^3^/s and 0~0.35 m/s, respectively. It can be seen from Figure 9b that despite the negative impact caused by velocity, flow rate is the determining factor and always positively affects WEC. This is because the flow rate (*Q*) is equals to the average velocity (*u*) multiplied by the discharge section area (*A*) and *A* grows with u for subcritical flow; consequently, *Q* grows at a faster rate than *u*. It can also be reflected in the rating curve between *Q* and *u* (Figure 7). Although the rise of *v* causes a decline in WEC, it also causes a rapid increase in *Q*, which may lead to a greater growth of WEC as a result. However, it should be noted that although *Q* and *u* were one-to-one correspondence based on the rating curve of Luanhe mainstream, they were assumed to be two independent variables in Figure 9b, i.e., the same *Q* could correspond to a different u because we regarded *A* as an arbitrary variable coefficient. Therefore, the maximum WEC with a high flow rate and low velocity might occur when *A* is very large (such as near the estuary); and the minimum WEC corresponds to a low flow rate and high velocity, which might occur with a small *A*, for example, in narrow mountainous rivers. It can be seen that no matter how the geomorphology of river channel changes, *Q* and *u* have similar effects on WEC.

### 4.3. Application of the Method to Multi-Segments and Management Implications

**Figure 10:**
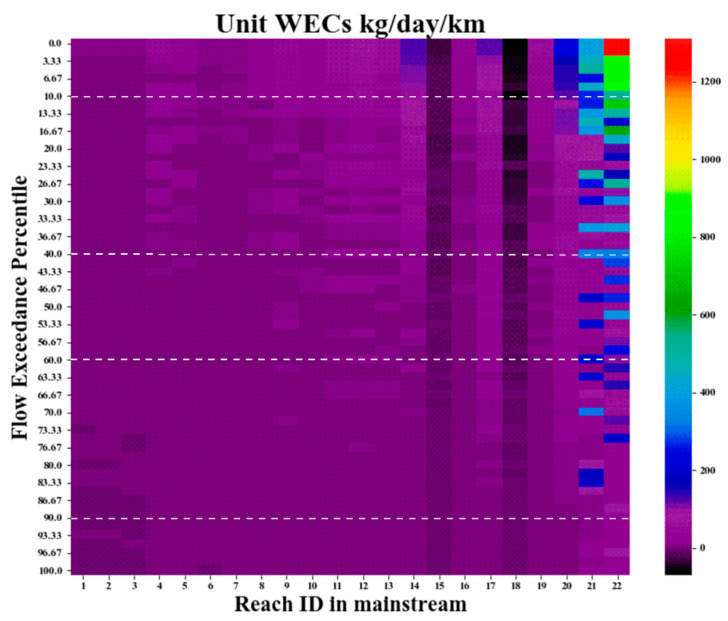
Estimation of unit WEC (kg/d/km) under different flow regimes in Luanhe Mainstream.

From the 17 subbasins created through SWAT in the Luanhe mainstream and administrative control units provided by the newly released “Fourteenth five-year plan” in China, we further demarcated the mainstream into 22 calculation units (labeled as Reach 1–Reach 22 from up to downstream, as shown in Figure 1b.). For comparison, we divided the element WECs by their reach lengths to convert them into unit WECs (kg/d/km), as shown in Figure 10. There was an apparent spatiotemporal variation of unit WECs from −65 to 1229 kg/day/km. The higher capacities were mainly concentrated downstream and during better flow conditions (i.e., upper right portion of the heat map), and there was little difference in unit WECs under 90% probability (i.e., low flow condition).

It should be noted that the WECs estimated in this study are ideal WECs, which do not consider any existing load discharge, point pollutants from sewage outlets, or non-point pollution from the riverside, for instance, and should reflect the impacts of nature conditions on water self-cleaning capacity [20]. However, the calculation of Reach 15 and Reach 18 showed a value below zero, which means that even though there is no pollutant discharge from the river bank (which is impossible), the water body cannot achieve the quality standard when it reaches the end section. This was derived from the inconsistent water quality criteria setting between the upstream (with looser standards) and downstream (with more stringent standards) of the two reaches. Based on the sampling data from Hadwen et al. [56], Zhao et al. [24] found the same circumstance of negative ideal WECs in two Australian rivers (Gwygir and Ovens Rivers). Actually, it can be explained that the allowable excessive discharge from upstream occupied the environmental capacity of lower reaches because of the self-contradictory settings of water quality standards. We therefore suggest cooperative management between Reach 14 and Reach 19. More specifically, the setting of the target water quality cannot only consider the local water functional plan, but the effects from adjacent areas. Furthermore, the maximum allowable loads in cooperative regions should be integrally allocated to sub-reaches according to the river length, water function plan, industrial structure, etc.

In the Luanhe mainstream, more than 77% of capacities were concentrated during HFI and MCI. The corresponding months were mainly from July to September, which accounted for 69% of the total WECs. The aggregation of allowable loads was because of the beneficial hydrodynamic conditions and relatively higher temperatures during the summer season. On the contrary, LFI was only 0.98% of the total WECs in the five years. This means that at least 90.2% capacities for NH_3_-N will be underestimated if we only take the LFI condition for WEC investigation as the guidance under CAEP [4]. Despite this conservative estimation, it is necessary for restoring and protecting the environment; however, excessive underestimation would also involve lots of unnecessary load reduction costs [8]. Comparing with the traditional LDC method in Figure 8b, our model more clearly reflects the effects of water temperature and velocity under different hydrological guarantee flow rate. To further verify the validity of our results, we compared our results with other studies in similar scenarios. Men et al. (2021) [57], also calculated the WEC in Luanhe mainstream, but only the part within Chengde city. This research was based on the traditional end-control WEC method, under the 90% hydrological guarantee flow rate and a constant integrated degradation coefficient. The result suggests that the WEC in Chende–Luanhe mainstream is 507.71 t/a (i.e., 1390.99 kg/d). In order to make a quantitative comparison, we extracted the results in our research involving in Chende, i.e., from Reach 2–Reach 17, and the five years average WEC in our dynamic method is 2039.92 kg/d. Because Men’s WEC model did not consider the variations of hydrological and temperature conditions, the result is usually a certain value, set as the upper limit of pollution discharge in water environmental management. It should be noticed that the control strategy in Men’s research is end-control, which has been proved as the most lenient strategy for pollutant discharging in the WEC management [25,29]. Nevertheless, it was still 31.81% lower than the WEC calculated by dynamic method. Dynamic treatment can control the discharge limit according to months or flow regimes, avoiding the unnecessary costs of load reduction. Li et al. (2022) [58] calculated the WEC in the Guojiatun–Wulongji section of Luanhe mainstream. Li’s research combining the MIKE11 model calculated the river WEC between June and July 2019. It also considered the dynamic changes of hydrological conditions and pollutant-integrated degradation coefficient through the MIKE11 model within a 30 s simulation step, and as a result, the monthly average WEC was 2550.33 kg/d. We extracted the results in June and July of Reach 5–Reach 14 to match Li’s research. The average WEC of our method was 2313.07 kg/d, fairly close to Li’s result. The comparisons further revealed the reliability and practicability of our method.

Meanwhile, during low flow periods, the dominant pollutant threat was from a point source, while nonpoint source pollutions appear in rainy seasons because of the stronger water flush processes. The allocation of WECs for these two pollution types should be considered separately in terms of different flow regimes [20,53,59]. Therefore, the unitary LFI consideration in current management may be inadequate, especially for non-point source diagnosis and control. For a more reasonable use of environmental resources in the whole basin, we suggest managers should separately control pollutant discharge following the distribution pattern of WECs in different flow regimes. Furthermore, site selection for discharge sources, such as poultry farms, wastewater treatment plants, and factories, might prefer regions with better self-cleaning capacities, i.e., Reach 20, Reach 21, and Reach 22. Factories able to cooperate with seasonal sewage discharge regulations can be planned for upper regions such as Reach 10–Reach 13. The head stream regions with lower ideal WECs (Reach 1–Reach 3 and Reach 6–Reach 7) need to be circumvented in factory site selection planning.

## 5. Conclusions

In this study, an integrated WEC estimation framework combining a modified hydro-environmental model and the LDC method developed a more appropriate pollutant control strategy for developing regions. The framework closely coupled the changes of hydrological condition and water temperature, dynamically presenting the spatiotemporal distribution of WECs under different hydrological regimes with low data. It can help local planners and managers identify hot spots for dischargeable regions and periods at a basin scale. The main findings can be summarized as follows:

(1) Hydrological conditions play a dominant role in WEC regulation. In the Luanhe River basin, 77% of the capacities were concentrated in high flows and moist conditions, and mainly from July to September. It would be more reasonable if the basin sewage discharge strategies were regulated according to different FDIs rather than a single low flow condition.
(2) The increase in flow velocity indeed promoted the decay rate of pollutants in the river, but shortened the traveling time within the calculated units, leading to the pollutants being washed downstream without adequate degradation, which eventually reduced the channel WEC.
(3) Considering the maldistribution of WECs between the upper and downstream, the point sources of pollution, e.g., sewage treatment plants, should be planned to avoid fragile regions of the upper and middle reaches.
(4) A coordinated water quality control should be implemented in the fragile reaches (e.g., Reach 15 and Reach 18 in Luanhe River), improving the quality standard of their adjacent upstream reaches to give space for local hydro-environmental restoration. Otherwise, the fragile reaches will hardly meet the standard, even if they have not discharged sewage.

Overall, the integrated framework presented a higher promotional value and applicability for developing regions’ loading capacities management. However, we did not quantitatively distinguish the point and non-point source loads, and only tentatively diagnosed them through the flow conditions based on their dominant periods. We hope that future SWAT models can further determine the quantified allocation of two pollutant sources. Furthermore, our model was not tested for low degradable pollutants, such as total phosphorus and heavy metals, which can alter aquatic community states by long-term persistence and interactions with other pollutants [60,61,62]. As a result, the applicability of our model should be further discussed when referring to these contaminants.

## Figures and Tables

**Table 1:** Performance rating (PR) for streamflow prediction by SWAT.

	Luanxian				Wulongji			
	Calibration	PR	Validation	PR	Calibration	PR	Validation	PR
R^2^	0.88	Very good	0.81	Very good	0.86	Very good	0.7	Good
NSE	0.81	Very good	0.64	Adequate	0.65	Very good	0.67	Very good

## Data Availability

Not applicable.
